# Stationary acceleration of Frenet curves

**DOI:** 10.1186/s13660-017-1354-7

**Published:** 2017-04-28

**Authors:** Nemat Abazari, Martin Bohner, Ilgin Sağer, Yusuf Yayli

**Affiliations:** 1 Department of Mathematics and Applications, University of Mohaghegh Ardabili, Ardabil, Iran; 2 Department of Mathematics and Statistics, Missouri University of Science and Technology, Rolla, Missouri 65409-0020 USA; 3 Department of Mathematics and Computer Science, University of Missouri - St. Louis, St. Louis, Missouri 63121 USA; 4 Department of Mathematics, Faculty of Science, Ankara University, Ankara, 06100 Turkey

**Keywords:** 53A17, 70G65, 53A04, stationary acceleration, spherical general helix, bi-invariant metric, Frenet elements, Minkowski space

## Abstract

In this paper, the stationary acceleration of the spherical general helix in a 3-dimensional Lie group is studied by using a bi-invariant metric. The relationship between the Frenet elements of the stationary acceleration curve in 4-dimensional Euclidean space and the intrinsic Frenet elements of the Lie group is outlined. As a consequence, the corresponding curvature and torsion of these curves are computed. In Minkowski space, for the curves on a timelike surface to have a stationary acceleration, a necessary and sufficient condition is refined.

## Introduction

Rigid body motion has attracted continuous attention since the time of Galileo and Bernoulli, and recently, the subject has generated a renewed interest in differential geometry. $\mathrm{SE}(3)$ is the space of all rigid body motions, and the motions can be described as curves in this space [1]. In 1989, Noakes, Heinzinger and Paden [2] derived the equations for the minimum acceleration curve by using positive definite bi-invariant metrics on the rotation group $\mathrm{SO}(3)$. By Noakes et al. [2], the specification of spline curves was extended to curves in groups associated with robotics. By using left-invariant metrics, Zefran and Kumar [3] in 1998 used the same acceleration definition for the rigid body as the covariant derivative of the motion, and so the jerk is the second covariant derivative. In 2007, Selig [4] repeated the analysis by using bi-invariant metrics on the rigid body motion group $\mathrm{SE}(3)$. Since these metrics are not positive definite, the curves specified by differential equations are derived only stationary, not minimal. In 1990, Bottema and Roth [5] studied a number of spatial motions by using the 4*D* representation of $\mathrm{SE}(3)$, one of which is the Serret-Frenet motion. Finding the curve with given curvature and torsion functions involves solving a system of differential equations given by the Serret-Frenet relations. This is not straightforward, and solutions are only known in a very few cases as studied by Lipkin [6] in 2005 and Selig [4] in 2007. In this work, the ideas of Zefran and Kumar [3] and Selig [4] are revisited. Kula et al., in [7], investigated the relations between a general helix and a slant helix. By using the Serret-Frenet frame in a 3-dimensional Lie group with a bi-invariant metric, the stationary acceleration of the spherical general helix is studied. It is proved that the normal curvature, geodesic curvature and geodesic torsion functions of the curves on a timelike surface in the Minkowsky space are linear.

## General helix in a Lie group

Let **G** be a 3-dimensional Lie group with the bi-invariant metric $\langle\cdot,\cdot \rangle$. Suppose ∇ is the corresponding Levi-Civita connection. If *g* denotes the Lie algebra of **G**, then the isomorphism $g\simeq T_{e}\mathbf{G}$ holds, where *e* is the identity element of **G**. As is known,

$$\bigl\langle X,[Y,Z] \bigr\rangle = \bigl\langle [X,Y],Z \bigr\rangle \quad \text{and}\quad \nabla_{X}Y=\frac{1}{2}[X,Y] $$

hold for all $X,Y,Z\in g$. Also, for any $X,Y\in g$, the vector product $X\times Y$ is defined by

$$\langle Z,X\times Y \rangle=\det(Z,X,Y) \quad\text{for all } Z\in g. $$

### Definition 2.1

General helix, see [8], Definition 1

Let $\alpha:I\to\mathbf{G}$ be a parameterized curve, where $I\subset{\mathbb {R}}$. Then *α* is called a *g*eneral helix if it makes a constant angle with a left-invariant vector field.

If $I\subset{\mathbb {R}}$ and $\alpha:I\to\mathbf{G}$ is a curve parameterized with arc length and if the Frenet structure of *α* is denoted by $(t,n,b,\kappa,\tau)$, then

$$\tau_{\mathbf{G}}=\frac{1}{2} \bigl\langle [t,n],b \bigr\rangle . $$

### Theorem 2.2

Lancret, see [8], Theorem 1

*A curve is a general helix in* **G** *if and only if* $\tau=c\kappa+\tau_{\mathbf{G}}$, *where* *c* *is a constant*.

### Definition 2.3

Left shift, see [8], Definition 3

Let $I\subset{\mathbb {R}}$ and $\alpha:I\to\mathbf{G}$ be an arc length parameterized curve. Then a curve $\beta:I\to g$, where *g* is the Lie algebra of **G**, for which $\beta'(s)=dL_{\alpha^{-1}(s)}\alpha'(s)$ for all $s\in I$, is called the *l*eft shift of *α*.

### Definition 2.4

Spherical curve, see [8], Definition 4

*α* is called a *s*pherical curve if *β* lies on the unit central sphere, i.e., $\langle\beta(s),\beta(s) \rangle=1$ for all $s\in I$.

### Theorem 2.5

See [8], Proposition 2

*A curve* *α* *is the spherical general helix with* $\tau=c\kappa+\tau_{\mathbf{G}}$ *if and only if*

$$\kappa(s)=\frac{1}{\sqrt{1-c^{2}s^{2}}},\qquad \tau(s)=\frac{c}{\sqrt{1-c^{2}s^{2}}}+ \tau_{\mathbf{G}}. $$

In the Lie group **G**, a spherical motion is determined by a unit speed space curve $\alpha(s)$. In the Serret-Frenet motion, a point on the moving body moves along the curve and the coordinate frame on the moving body remains aligned with the tangent *t*, normal *n* and bi-normal *b* of the curve. Using the 4*D* representation of *G*, the motion can be specified as

2.1 $$ G(s)= \begin{pmatrix} R(s) & \alpha(s)\\ 0 & 1 \end{pmatrix} , $$

where *α* is the curve, and the rotation matrix has the unit vectors *t*, *n* and *b* as columns of

$$R= (t\mid n\mid b ). $$

Set $\nabla_{t}x=x'$ for all $x\in\{t,n,b\}$. Now the intrinsic Serret-Frenet formulas are

$$t'=\kappa n,\qquad n'=-\kappa t+\tau b, \qquad b'=-\tau n, $$

where *κ* and *τ* are the curvature and torsion functions of the curve, respectively. The Darboux vector $\omega=\tau t+\kappa b$ has the properties

$$t'=\omega\times t,\qquad n'=\omega\times b,\qquad b'=\omega\times n, $$

see [6], Section 10.2. This means that

$$R'=\Omega_{\omega}R $$

can be written for the $3\times3$ anti-symmetric matrix $\Omega _{\omega}$, which is corresponding to *ω*. Since *α* is a unit speed curve, we have $\alpha'=t$, and hence

$$V=G'G^{-1} = \begin{pmatrix} \Omega_{\omega} & t-\omega\times\alpha\\ 0 & 0 \end{pmatrix} . $$

Using the Serret-Frenet relations, the derivative of the velocity can be calculated as

$$V'= \begin{pmatrix} \Omega_{\omega'} & t'-\omega'\times\alpha\\ 0 & 0 \end{pmatrix} , $$

where $\omega'=\tau't+\kappa'b$. Hence, the second derivative of the velocity is

$$V''= \begin{pmatrix} \Omega_{\omega''} & -\omega''\times\alpha-\kappa'n\\ 0 & 0 \end{pmatrix} , $$

where $n=b\times t$ and $\omega''=\tau''t+(\tau'\kappa-\kappa'\tau)n+\kappa''b$. Finally, $G^{-1}V''G$ is computed as

2.2 $$ G^{-1}V''G = \begin{pmatrix} 0 & -\kappa'' & \tau'\kappa-\kappa'\tau& 0\\ \kappa'' & 0 & -\tau'' & -\kappa'\\ \kappa'\tau-\tau'\kappa& \tau'' & 0 & 0\\ 0 & 0 & 0 & 0 \end{pmatrix} . $$

Since the curve *α* is a stationary acceleration curve,

$$G^{-1}V''G=C $$

holds, see [4]. Thus, there exist constants $c_{1}$, $c_{2}$, $c_{3}$, $c_{4}$ such that

2.3 $$ G^{-1}V''G=C = \begin{pmatrix} 0 & -c_{1} & c_{2} & 0\\ c_{1} & 0 & -c_{3} & -c_{4}\\ -c_{2} & c_{3} & 0 & 0\\ 0 & 0 & 0 & 0 \end{pmatrix} . $$

By setting up this equation for the two unknowns *κ* and *τ*, the system of differential equations

$$\kappa''=c_{1},\qquad \tau' \kappa-\kappa'\tau=c_{2},\qquad \tau''=c_{3}, \qquad\kappa'=c_{4} $$

holds, and as a consequence, the following theorem is true.

### Theorem 2.6

*The general spherical helix is a stationary acceleration curve in a Lie group* **G** *with the bi*-*invariant metric if and only if* $\kappa=1$ *and* *τ* *is linear*.

### Proof

Let the curve *α* be a general spherical helix in the Lie group **G**. Then $\tau=c\kappa+\tau_{\mathbf{G}}$, where *c* is a constant. *α* is a stationary acceleration curve, and due to (2.2) and (2.3), ${\kappa}'=c_{4}$ is satisfied. Therefore, $\kappa=c_{4}s+c_{5}$, and from $\kappa(s)=\frac{1}{\sqrt{1-c^{2}s^{2}}}$, $c=0$ is obtained. Hence, $\kappa=1$, and so $c_{2}=\tau'\kappa-\kappa'\tau=\tau '_{\mathbf{G}}$. Therefore, $\tau_{\mathbf{G}}=c_{2}s+c_{6}$, where $c_{6}$ is a constant. Finally, $\tau=c+\tau_{\mathbf{G}}=a+bs$, where $a=c+c_{6}$ and $b=c_{2}$ are constants and $\tau''=0$. Hence, $\kappa=1$ and *τ* is linear. Conversely, if $\kappa=1$ and *τ* is linear, then obviously *κ* and *τ* satisfy the stationary acceleration curve condition (2.2). □

## Spherical general helix

For $I\subset{\mathbb {R}}$, $\alpha:I\to S^{3}$, the unit sphere with the center at origin in ${\mathbb {R}}^{4}$ is an immersed curve in a 3-dimensional real space form. Therefore, any curve on $S^{3}$ can also be considered to be a curve in ${\mathbb {R}}^{4}$. In this paper, the goal is to obtain the relationship between the Frenet frame $(e_{1}\mid e_{2}\mid e_{3}\mid e_{4})$ in 4-dimensional Euclidean space with the curvature functions $k_{i}= \langle e'_{i},e_{i+1} \rangle$ for $i=1,2,3$ and the intrinsic Frenet frame $(t\mid n\mid b)$ with the curvature $\kappa= \langle t',n \rangle$ and torsion $\tau= \langle n',b \rangle$ of the curve *α*. Set $t=e_{1}$. By using the Gauss map of the sphere,

3.1 $$ \kappa=\sqrt{k_{1}^{2}-1} $$

and

3.2 $$ \tau=\frac{k_{1}^{2}k_{2}}{\kappa^{2}} $$

hold, see [9].

### Theorem 3.1

*A unit speed space curve* $\alpha:I\subset{\mathbb {R}}\to S^{3}$ *parameterized with arc length is a stationary acceleration curve if and only if*

$$k_{1}=\pm\sqrt{(as+b)^{2}+1} $$

*and*

$$k_{2}=\frac{(as+b)^{2}(ps+q)}{(as+b)^{2}+1}, $$

*where* *a*, *b*, *p*, *q* *are constants and* $k_{i}$ *is the* *ith principle curvature of the curve* *α* *for* $i=1,2$ *in* 4-*dimensional Euclidean space*.

### Proof

Suppose *α* is a unit speed space curve on $S^{3}$. It is known that, in the Frenet-Serret motion, a point on the moving body moves along the curve *α* and the coordinate frame in the moving body remains aligned with the tangent *t*, normal *n* and bi-normal *b* of this curve. By using the 4D representation, the motion can be specified in the form (2.1) such that the corresponding rotation matrix of motion is $R= (t \vert n \vert b )$. The curve *α* is a stationary acceleration curve if and only if $G^{-1}V''G=C$, where *C* is the $4\times4$ constant matrix as in (2.3). By substituting $k_{1}=x$ in (3.1), $\kappa=\sqrt{x^{2}-1} $ is obtained, and from (3.1), we get $\kappa'=c_{4}$. From

$$\kappa'=c_{4},\qquad\tau''=c_{3}, \qquad \tau'\kappa-\kappa'\tau=c_{2}, $$

we have

$$c_{2} =\tau'\kappa-c_{4}\tau\quad\text{and} \quad 0=\tau''\kappa+\tau' \kappa'-c_{4}\tau'=c_{3}\kappa. $$

Since $\kappa\neq0$, we get $c_{3}=0$. Hence,

$$\kappa=c_{4}s+b \quad\text{and}\quad \tau=ps+q. $$

On the other hand, it is clear that

3.3 $$ \kappa=\sqrt{k_{1}^{2}-1} \quad\text{and}\quad \tau=\frac{k_{1}^{2}k_{2}}{k^{2}} $$

are satisfied. Therefore, for $c_{4}=a$, we obtain

3.4 $$ k_{1}=\pm\sqrt{\kappa^{2}+1}=\pm \sqrt{(as+b)^{2}+1} $$

and

3.5 $$ k_{2}=\frac{\tau\kappa^{2}}{k_{1}^{2}}=\frac{\tau\kappa^{2}}{\kappa^{2}+1} = \frac{(as+b)^{2}(ps+q)}{(as+b)^{2}+1}, $$

and by using $c_{3}=0$, we get

$$G^{-1}V''G=C = \begin{pmatrix} 0 & 0 & c_{2} & 0\\ 0 & 0 & 0 & -c_{4}\\ -c_{2} & 0 & 0 & 0\\ 0 & 0 & 0 & 0 \end{pmatrix} , $$

where *C* is a $4\times4$ constant matrix such that $k_{1}$ and $k_{2}$ satisfy the stationary acceleration condition of *α*. Conversely, if $k_{1}$ and $k_{2}$ satisfy (3.4) and (3.5), then, from (3.1) and (3.2), we have $\kappa=as+b$ and $\tau=ps+q$. Thus, from (2.2), $G^{-1}V''G$ is a $4\times4$ constant matrix, and so *α* is a stationary acceleration curve. □

## Curves on a timelike surface

The Minkowski spacetime ${\mathbb {R}}^{3}_{1}$ is the Euclidean space ${\mathbb {R}}^{3}$ with the inner product

$$\langle x,y \rangle=-x_{1}y_{1}+x_{2}y_{2}+x_{3}y_{3}, \quad\text{where } x=(x_{1},x_{2},x_{3}),y=(y_{1},y_{2},y_{3}) \in{\mathbb {R}}^{3}. $$

A vector $v\in{\mathbb {R}}^{3}_{1}\setminus\{0\}$ is spacelike, timelike or lightlike if $\langle v,v \rangle>0$, $\langle v,v \rangle<0$ or $\langle v,v \rangle=0$. The vector $v=0$ is spacelike. Also, the norm of a vector *v* is given by $\Vert v \Vert =\sqrt{ \vert \langle v,v \rangle \vert }$.

Let $X:U\to{\mathbb {R}}^{3}_{1}$ be a timelike embedding, where *U* is an open subset of ${\mathbb {R}}^{2}$. The tangent space $T_{p}M$ is a timelike plane at any $p\in X(U)$, where $M=X(U)$. Let $\overline{\gamma}:I\to U$ be a regular curve and define the curve $\gamma:I\to M\subset{\mathbb {R}}^{3}_{1}$ on the timelike surface by $\gamma(s)=X(\overline{\gamma})$. Let *γ* be spacelike or timelike on the timelike surface *M* with the unit tangent vector $t(s)=\gamma'(s)$, where *s* is the arc-length parameter. Since $M=X(U)$ is timelike, a unit spacelike normal vector field *n* on $M=X(U)$ is defined by

$$n(p)=\frac{X_{u_{1}}(u)\times X_{u_{2}}(u)}{ \Vert X_{u_{1}}(u)\times X_{u_{2}}(u) \Vert } \quad\text{for all } p=X(u). $$

Then $n_{\gamma}=n\circ\gamma$ is a unit spacelike normal vector field along *γ*. The bi-normal vector field is defined by $(\varepsilon\circ\gamma)b=n_{\gamma}\times t$. It is known that

$$\langle t,t \rangle=\varepsilon\circ\gamma,\qquad \langle n_{\gamma},n_{\gamma} \rangle=1,\qquad \langle n_{\gamma},b \rangle=0,\qquad \langle b,b \rangle=-(\varepsilon\circ\gamma), $$

where $\varepsilon\circ\gamma=\operatorname{sgn}(t)$ which equals 1 when *γ* is spacelike and equals −1 when *γ* is timelike. When $\varepsilon(\gamma(s))=1$, the semi-orthonormal frame is $(b(s)\mid n_{\gamma}(s)\mid t(s) )$, and when $\varepsilon(\gamma(s))=-1$, the semi-orthonormal frame is $(t(s)\mid b(s)\mid n_{\gamma }(s) )$. Therefore, we have

$$b(s)\times n_{\gamma}(s)=-\varepsilon \bigl(\gamma(s) \bigr)t(s) \quad \text{and}\quad t(s)\times b(s)=-n_{\gamma}. $$

For

$$k_{n}(s)= \bigl\langle n_{\gamma}(s),t'(s) \bigr\rangle ,\qquad k_{g}(s)= \bigl\langle b(s),t'(s) \bigr\rangle ,\qquad \tau_{g}(s)= \bigl\langle n_{\gamma}(s),b'(s) \bigr\rangle , $$

the Frenet equations are

$$\begin{aligned} t' =& -(\varepsilon\circ\gamma)k_{g}b+k_{n}n_{\gamma}, \qquad n_{\gamma}'=(\varepsilon\circ\gamma) \tau_{g}b-( \varepsilon\circ \gamma)k_{n}t, \\ b' =& \tau_{g}n_{\gamma}-(\varepsilon\circ\gamma)k_{g}t, \end{aligned}$$

and these are called normal curvature, geodesic curvature and geodesic torsion, respectively [9]. Now we suppose $t'(s)\neq0$. The Darboux vector field in two cases $\varepsilon(\gamma(s))=\pm1$ is

$$\omega(s)=-\tau_{g}(s)t(s)-k_{g}(s)n_{\gamma}(s)+k_{n}(s)b(s). $$

Therefore,

$$\omega\times t=t',\qquad \omega\times n_{\gamma}=n_{\gamma}', \qquad \omega\times b=b'. $$

Also, we have

$$\omega'=-\tau_{g}'t-k_{g}'n_{\gamma}+k_{n}'b $$

and

$$\begin{aligned} \omega'' =& \bigl(-\tau_{g}''+( \varepsilon\circ\gamma)k_{g}'k_{n} -( \varepsilon\circ\gamma)k_{n}'k_{g} \bigr)t \\ &{}+ \bigl(-k_{g}''-\tau_{g}'k_{n}+k_{n}' \tau_{g} \bigr)n_{\gamma} \\ &{}+ \bigl(k_{n}''+(\varepsilon\circ\gamma) \tau_{g}'k_{g} -(\varepsilon\circ \gamma)k_{g}'\tau_{g} \bigr)b \\ =& A_{1}t+A_{2}n_{\gamma}+A_{3}b, \end{aligned}$$

where

$$\begin{aligned} A_{1} =&-\tau_{g}''+(\varepsilon \circ\gamma)k_{g}'k_{n}-(\varepsilon\circ \gamma)k_{n}'k_{g}, \\ A_{2} =&-k_{g}''- \tau_{g}'k_{n}+k_{n}' \tau_{g}, \\ A_{3} =&k_{n}''+(\varepsilon\circ \gamma)\tau_{g}'k_{g}-(\varepsilon\circ \gamma)k_{g}'\tau_{g}. \end{aligned}$$

Let $\Omega_{\omega}$ be the anti-symmetric 3×3 matrix corresponding to the Darboux vector field *ω*, so

$$\Omega_{\omega} = \begin{pmatrix} 0 & -k_{n} & -k_{g}\\ k_{n} & 0 & \tau_{g}\\ k_{g} & -\tau_{g} & 0 \end{pmatrix} $$

and

$$\Omega_{\omega''} = \begin{pmatrix} 0 & -A_{3} & A_{2}\\ A_{3} & 0 & -A_{1}\\ -A_{2} & A_{1} & 0 \end{pmatrix} . $$

By using the 4D representation of $\mathrm{SE}(3)$, the motion can be specified as

$$G(s) = \begin{pmatrix} R(s) & \gamma(s)\\ 0 & 0 \end{pmatrix} , $$

where

$$R(s)= \bigl(t(s)\mid b(s)\mid n_{\gamma}(s) \bigr) \quad\text{if } \varepsilon \bigl(\gamma(s) \bigr)=-1 $$

and

$$R(s)= \bigl(b(s)\mid n_{\gamma}(s)\mid t(s) \bigr) \quad\text{if } \varepsilon \bigl(\gamma(s) \bigr)=+1. $$

From the properties of the Darboux vector, we can write $R'=\Omega _{\omega}R$. Hence, we have

$$V=G'G^{-1} = \begin{pmatrix} \Omega_{\omega} & t-\omega\times\gamma\\ 0 & 0 \end{pmatrix} . $$

Therefore,

$$\begin{aligned} V'' =& \begin{pmatrix} \Omega_{\omega''} & -\omega''\times\gamma-\omega'\times t\\ 0 & 0 \end{pmatrix} \\ =& \begin{pmatrix} \Omega_{\omega''} & -\omega''\times\gamma-k'_{g}(\varepsilon\circ\gamma)b-k_{n}'n_{\gamma }\\ 0 & 0 \end{pmatrix} , \end{aligned}$$

and thus

$$\begin{aligned} G^{-1}V''G =& \begin{pmatrix} R^{T} & -R^{T}\gamma\\ 0 & 1 \end{pmatrix} \begin{pmatrix} \Omega_{\omega''} & -\omega''\times\gamma-k'_{g}(\varepsilon\circ\gamma)b-k_{n}'n_{\gamma }\\ 0 & 0 \end{pmatrix} \begin{pmatrix} R & \gamma\\ 0 & 1 \end{pmatrix} \\ =& \begin{pmatrix} R^{T}\Omega_{\omega''}R & -k_{n}'R^{T}n_{\gamma}-(\varepsilon\circ\gamma)k_{g}'R^{T}b\\ 0 & 0 \end{pmatrix} . \end{aligned}$$

By using the standard formulas for the scalar and vector products of *t*, $n_{\gamma}$ and *b*, we can write

$$G^{-1}V''G = \begin{pmatrix} 0 & -A_{3} & A_{2} & 0\\ A_{3} & 0 & -A_{1} & -k_{n}'\\ -A_{2} & A_{1} & 0 & -(\varepsilon\circ\gamma)k_{g}'\\ 0 & 0 & 0 & 0 \end{pmatrix} , $$

where $A_{1}$, $A_{2}$ and $A_{3}$ are as mentioned above. From $G^{-1}V''G=C$, where *C* is a $4\times4$ constant matrix, we obtain

$$k_{n}'(s)=c_{1} \quad\text{and}\quad k_{g}'(s)=c_{2}. $$

Then

$$k_{n}(s)=c_{1}s+a_{1},\qquad k_{g}(s)=c_{2}s+a_{2},\qquad k_{g}''(s)=0, \qquad k_{n}''(s)=0. $$

Also, from

$$\begin{aligned} -\tau_{g}''(s)+\varepsilon \bigl(\gamma(s) \bigr)k_{g}'(s)k_{n}(s)-\varepsilon \bigl( \gamma (s) \bigr)k_{n}'(s)k_{g}(s) =&c_{3}, \\ -\tau_{g}'(s)k_{n}(s)+k_{n}'(s) \tau_{g}(s) =&c_{4}, \\ \varepsilon \bigl(\gamma(s) \bigr)\tau_{g}'(s)k_{g}(s)- \varepsilon \bigl(\gamma (s) \bigr)k_{g}'(s) \tau_{g}(s) =&c_{5}, \end{aligned}$$

we can obtain $\tau_{g}(s)=as+b$. Hence, we have the following result.

### Theorem 4.1

*A curve on the timelike surface in Minkowski space is a stationary acceleration curve if and only if its normal curvature*, *geodesic curvature and geodesic torsion are linear functions*.

## Curves on Minkowski spacetime

The Minkowski spacetime ${\mathbb {R}}^{4}_{1}$ is the Euclidean space ${\mathbb {R}}^{4}$ with the inner product

$$\langle x,y \rangle=-x_{1}y_{1}+x_{2}y_{2}+x_{3}y_{3}+x_{4}y_{4}, \quad\text{where } x=(x_{1},x_{2},x_{3},x_{4}),y=(y_{1},y_{2},y_{3},y_{4}) \in{\mathbb {R}}^{4}. $$

A vector $v\in{\mathbb {R}}^{4}_{1}\setminus\{0\}$ is spacelike, timelike or lightlike if $\langle v,v \rangle>0$, $\langle v,v \rangle<0$ or $\langle v,v \rangle=0$. The vector $v=0$ is spacelike. Also, the norm of a vector *v* is given by $\Vert v \Vert =\sqrt{ \vert \langle v,v \rangle \vert }$. Let *α* be a unit speed timelike or spacelike curve with $(e_{1}\mid e_{2}\mid e_{3}\mid e_{4} )$ as the Frenet frame in ${\mathbb {R}}^{4}_{1}$ and set $\langle e_{i},e_{i} \rangle=\varepsilon_{i}\in\{-1,1\} $, $i=1,2,3,4$. We can define the curvature functions by $k_{i}= \langle e'_{i},e_{i+1} \rangle$ for $i=1,2,3$. Therefore, the Frenet equations are

$$ \begin{pmatrix} e_{1}'\\ e_{2}'\\ e_{3}'\\ e_{4}' \end{pmatrix} = \begin{pmatrix} 0 & k_{1}\varepsilon_{2} & 0 & 0\\ -k_{1}\varepsilon_{1} & 0 & k_{2}\varepsilon_{2} & 0\\ 0 & -k_{2}\varepsilon_{2} & 0 & -k_{3}\varepsilon_{1}\varepsilon _{2}\varepsilon_{3}\\ 0 & 0 & -k_{3}\varepsilon_{3} & 0 \end{pmatrix} \begin{pmatrix} e_{1}\\ e_{2}\\ e_{3}\\ e_{4} \end{pmatrix} . $$

Also, the vector $x\times y\times z$ is defined by

$$x\times y\times z =\left \vert \begin{matrix} -i & j & k & l\\ x_{1} & x_{2} & x_{3} & x_{4}\\ y_{1} & y_{2} & y_{3} & y_{4}\\ z_{1} & z_{2} & z_{3} & z_{4} \end{matrix} \right \vert , $$

here $\{i,j,k,l\}$ is the canonical basis of ${\mathbb {R}}^{4}_{1}$ and

$$x=(x_{1},x_{2},x_{3},x_{4}),\qquad y=(y_{1},y_{2},y_{3},y_{4}),\qquad z=(z_{1},z_{2},z_{3},z_{4}) \in{\mathbb {R}}^{4}. $$

Then, for any $t\in{\mathbb {R}}^{4}_{1}$, we can write $\langle t,x\times y\times z \rangle=\det(t,x,y,z)$. Thus, $x\times y\times z$ is semi-orthogonal to *x*, *y* and *z*. A normal curve in ${\mathbb {R}}^{4}_{1}$ is a curve whose position vector always lies in its normal space $e^{\perp}_{1}=\{w\in{\mathbb {R}}^{4}_{1}: \langle w,e_{1} \rangle=0\}$.

### Theorem 5.1

See [10], Theorem 3.1

*Let* *α* *be a unit speed timelike or spacelike curve with non*-*lightlike vector fields* $e_{2}$, $e_{3}$, $e_{4}$, *lying in* ${\mathbb {R}}^{4}_{1}$. *Then* *α* *is congruent to a normal curve if and only if*

$$\frac{k_{3}\varepsilon_{3}}{k_{2}} \biggl(\frac{1}{k_{1}} \biggr)' = \varepsilon_{1} \biggl[\frac{1}{k_{3}} \biggl(\frac{k_{2}}{k_{1}}+ \varepsilon _{2}\varepsilon_{3} \biggl( \biggl( \frac{1}{k_{1}} \biggr)'\frac{1}{k_{2}} \biggr)' \biggr) \biggr]'. $$

### Theorem 5.2

See [10], Formula (3.3)

*Let* *α* *be a unit speed timelike or spacelike normal curve with non*-*lightlike vector fields* $e_{2}$, $e_{3}$, $e_{4}$, *lying in* ${\mathbb {R}}^{4}_{1}$. *Then its position vector satisfies the equation*

$$\alpha = -\frac{\varepsilon_{1}}{k_{1}}e_{2}-\frac{\varepsilon_{1}\varepsilon_{2}}{k_{2}} \biggl( \frac{1}{k_{1}} \biggr)'e_{3} -\frac{\varepsilon_{1}}{k_{3}} \biggl(\frac{k_{2}}{k_{1}}+\varepsilon _{2}\varepsilon_{3} \biggl( \biggl(\frac{1}{k_{1}} \biggr)'\frac{1}{k_{2}} \biggr)' \biggr)e_{4}. $$

Let *M* be a hypersurface in ${\mathbb {R}}^{4}_{1}$ with the induced Levi-Civita connection ∇ of ${\mathbb {R}}^{4}_{1}$. Let $\alpha:I\to M$ be a non-lightlike immersed unit speed curve in *M*, and let us denote the Frenet frame by $(t\mid n\mid b)$. The Frenet equations are

$$t'=\nabla_{t}t=\kappa n,\qquad n'= \nabla_{t}n=\varepsilon_{b}\kappa t+\tau b,\qquad b'=\nabla_{t} b=\varepsilon_{t}\tau n, $$

where $\varepsilon_{X}= \langle X,X \rangle$ and *κ*, *τ* are curvature and torsion functions, respectively, and *t*, *n*, *b* satisfy the equations

$$b\times n=\varepsilon_{t}t,\qquad t\times b=\varepsilon_{n}n, \qquad n\times t=\varepsilon_{b} b. $$

The Darboux vector field is $\omega=-\varepsilon_{b}\tau t-\varepsilon _{n}\kappa b$. Therefore, we have

$$\omega\times t=t',\qquad \omega\times n=n',\qquad \omega \times b=b'. $$

Also, we have

$$\omega'=-\varepsilon_{b}\tau't- \varepsilon_{n}\kappa'b $$

and

$$\omega'' =-\varepsilon_{b} \tau''t-\varepsilon_{b} \bigl( \tau'\kappa-\kappa'\tau \bigr)n-\varepsilon_{n} \kappa''b. $$

If $R=(t\mid n\mid b)$ is the rotation matrix and $\Omega_{\omega}$ is the corresponding $3\times3$ anti-symmetric matrix to the Darboux vector *ω*, then

$$\Omega_{\omega''} = \begin{pmatrix} 0 & \varepsilon_{n}\kappa'' & -\varepsilon_{b}(\tau'\kappa-\kappa '\tau)\\ -\varepsilon_{n}\kappa'' & 0 & \varepsilon_{b}\tau''\\ \varepsilon_{b}(\tau'\kappa-\kappa'\tau) & -\varepsilon_{b}\tau'' & 0 \end{pmatrix} , $$

where $\alpha'=t$. Therefore, for the motion

$$G(s)= \begin{pmatrix} R(s) & \alpha(s)\\ 0 & 1 \end{pmatrix} , $$

from the properties of the Darboux vector, we have

$$R'=\Omega R \quad\text{and}\quad V=G'G^{-1}. $$

Hence,

$$V'' = \begin{pmatrix} \Omega_{\omega''} & -\omega''\times\alpha-\omega'\times t\\ 0 & 0 \end{pmatrix} = \begin{pmatrix} \Omega_{\omega''} & -\omega''\times\alpha-\varepsilon_{n}\kappa'n\\ 0 & 0 \end{pmatrix} $$

and

$$G^{-1}V''G = \begin{pmatrix} 0 & \varepsilon_{n}\kappa'' & -\varepsilon_{b}(\tau'\kappa-\kappa '\tau) & 0\\ -\varepsilon_{n}\kappa'' & 0 & \varepsilon_{b}\tau'' & -\varepsilon _{n}\kappa'\\ \varepsilon_{b}(\tau'\kappa-\kappa'\tau) & -\varepsilon_{b}\tau'' & 0 & 0\\ 0 & 0 & 0 & 0 \end{pmatrix} =C, $$

where *C* is a $4\times4$ constant matrix, which is the necessary and sufficient condition for non-lightlike immersed curves $\alpha:I\to M$ in the hypersurface $M\subset{\mathbb {R}}^{4}_{1}$ to be an acceleration curve. Then, by $G^{-1}V''G=C$, we obtain

$$\kappa=as+b \quad\text{and}\quad \tau=ps+q, $$

where *a*, *b*, *p*, *q* are constants and *s* is the arc-length parameter of the curve *α*.

Let $(e_{1}\mid e_{2}\mid e_{3}\mid e_{4})$ be the Frenet frame of the normal curve *α* as a unit speed timelike or spacelike normal curve with non-lightlike vector fields $e_{2}$, $e_{3}$, $e_{4}$, lying in ${\mathbb {R}}^{4}_{1}$. Let the curvature functions of *α* be $k_{1}$, $k_{2}$, $k_{3}$. Then

$$t'=e_{1}'-\varepsilon \bigl\langle e_{1}',\alpha \bigr\rangle \alpha =\varepsilon_{2}k_{1} \bigl(e_{2}-\varepsilon \langle e_{2},\alpha \rangle\alpha \bigr), $$

where $\langle v,v \rangle=\varepsilon$ with *v* the unit normal vector field to the hypersurface *M* in ${\mathbb {R}}^{4}_{1}$ and $\langle e_{i},e_{i} \rangle=\varepsilon_{i}\in\{-1,1\}$, $i=1,2,3,4$. Also, by using the Gauss map [11] and Theorem 5.1, we can write

$$n=\frac{t'}{ \Vert t' \Vert } =\frac{e_{2}-\varepsilon \langle e_{2},\alpha \rangle\alpha}{ \sqrt{1-\varepsilon \langle e_{2},\alpha \rangle^{2}}}, $$

and from Theorem 5.2, we can write

$$\kappa= \bigl\langle t',n \bigr\rangle =k_{1}\sqrt{1- \varepsilon \langle e_{2},\alpha \rangle^{2}} =\sqrt {k^{2}_{1}-\varepsilon}. $$

The bi-normal vector is

$$\alpha\times t\times n =\frac{k_{1}}{\sqrt{k^{2}_{1}-\varepsilon}}\alpha\times e_{1}\times e_{2} =\frac{1}{\sqrt{1-\frac{\varepsilon}{k^{2}_{1}}}}\alpha\times e_{1}\times e_{2}. $$

Therefore,

$$b'= \biggl(\frac{1}{\sqrt{1-\frac{\varepsilon}{k^{2}_{1}}}} \biggr)'\alpha \times e_{1}\times e_{2} +\frac{\varepsilon_{2}k_{2}}{\sqrt{1-\frac{\varepsilon}{k^{2}_{1}}}}\alpha \times e_{1}\times e_{3}. $$

Then $\langle b',\alpha \rangle=0$. Finally,

$$\begin{aligned} \tau =& - \bigl\langle b',n \bigr\rangle =- \biggl\langle \frac{\varepsilon_{3}k_{2}}{\sqrt{1-\frac{\varepsilon }{k^{2}_{1}}}}\alpha \times e_{1}\times e_{3}, \frac{1}{\sqrt{1-\varepsilon { \langle e_{2},\alpha \rangle}^{2}}}e_{2} \biggr\rangle \\ =& - \biggl\langle \frac{\varepsilon_{3}k_{2}}{\sqrt{1-\frac {\varepsilon}{k^{2}_{1}}}}\alpha \times e_{1}\times e_{3},\frac{1}{\sqrt {1-\frac{\varepsilon}{k^{2}_{1}}}}e_{2} \biggr\rangle \\ =& -\frac{\varepsilon_{3}k_{2}}{1-\frac{\varepsilon}{k^{2}_{1}}} \langle\alpha\times e_{1}\times e_{3},e_{2} \rangle \\ =& \frac{\varepsilon_{3}k_{2}k^{2}_{1}}{\kappa^{2}}. \end{aligned}$$

Hence, we have proved the following result.

### Theorem 5.3

*Let* *M* *be a hypersurface in* ${\mathbb {R}}^{4}_{1}$ *and* $I\subset{\mathbb {R}}$. *Let* $\alpha:I\to M$ *be a unit speed timelike or spacelike normal curve with non*-*lightlike vector fields* $e_{2}$, $e_{3}$, $e_{4}$, *lying in* ${\mathbb {R}}^{4}_{1}$. *Then* *α* *is an acceleration curve in* *M* *if and only if*

$$k_{1}=\pm\sqrt{(as+b)^{2}+\varepsilon} $$

*and*

$$k_{2}=\frac{\varepsilon_{3}(as+b)^{2}(ps+q)}{(as+b)^{2}+\varepsilon}, $$

*where* *a*, *b*, *p*, *q* *are constants and* $k_{i}$ *is the* *ith principle curvature of the curve* *α* *for* $i=1,2$ *in* ${\mathbb {R}}^{4}_{1}$ *and* $\langle v,v \rangle=\varepsilon$ *with* *v* *the unit normal vector field to the hypersurface* *M* *in* ${\mathbb {R}}^{4}_{1}$ *and* $\langle e_{3},e_{3} \rangle=\varepsilon_{3}$.

## Results and discussion

In this paper, it is proved that the general spherical helix is the stationary acceleration curve in a Lie group with a bi-invariant metric if and only if its curvature is unit and torsion is linear. The relationship between the Frenet elements of the stationary acceleration curve in 4-dimensional Euclidean space and the intrinsic Frenet elements of the Lie group is obtained. In other words, the necessary and sufficient conditions for stationary acceleration of unit speed spherical curves are studied, and as a consequence, the corresponding curvature and torsion of these curves are derived.
